# Tomatidine, a natural steroidal alkaloid shows antiviral activity towards chikungunya virus *in vitro*

**DOI:** 10.1038/s41598-020-63397-7

**Published:** 2020-04-14

**Authors:** Berit Troost, Lianne M. Mulder, Mayra Diosa-Toro, Denise van de Pol, Izabela A. Rodenhuis-Zybert, Jolanda M. Smit

**Affiliations:** 1 Department of Medical Microbiology and Infection Prevention, University of Groningen; University Medical Center Groningen, Groningen, the Netherlands; 2 Present Address: Programme in Emerging Infectious Diseases, Duke-NUS Medical School, Singapore, 169857 Singapore

**Keywords:** Alphaviruses, Antivirals

## Abstract

In recent decades, chikungunya virus (CHIKV) has re-emerged, leading to outbreaks of chikungunya fever in Africa, Asia and Central and South America. The disease is characterized by a rapid onset febrile illness with (poly)arthralgia, myalgia, rashes, headaches and nausea. In 30 to 40% of the cases, CHIKV infection causes persistent (poly)arthralgia, lasting for months or even years after initial infection. Despite the drastic re-emergence and clinical impact there is no vaccine nor antiviral compound available to prevent or control CHIKV infection. Here, we evaluated the antiviral potential of tomatidine towards CHIKV infection. We demonstrate that tomatidine potently inhibits virus particle production of multiple CHIKV strains. Time-of -addition experiments in Huh7 cells revealed that tomatidine acts at a post-entry step of the virus replication cycle. Furthermore, a marked decrease in the number of CHIKV-infected cells was seen, suggesting that tomatidine predominantly acts early in infection yet after virus attachment and cell entry. Antiviral activity was still detected at 24 hours post-infection, indicating that tomatidine controls multiple rounds of CHIKV replication. Solasodine and sarsasapogenin, two structural derivatives of tomatidine, also showed strong albeit less potent antiviral activity towards CHIKV. In conclusion, this study identifies tomatidine as a novel compound to combat CHIKV infection *in vitro*.

## Introduction

Chikungunya virus (CHIKV) was first identified in Tanzania between 1952 and 1953, where it caused an outbreak of chikungunya fever described as febrile polyarthralgia^1^. Within the last decade, CHIKV re-emerged and caused various large outbreaks in Africa, Asia and Central and South America^2,3^. Among those was an explosive epidemic on the Indian Ocean island of La Reunion, where over 266.000 people were affected^4^. Furthermore, since its introduction in the Americas in 2013, CHIKV has spread to over 45 countries in Central and South America causing more than 1.7 million infections^5^.

CHIKV is transmitted to humans via the mosquito vectors *Aedes aegypti* and *Aedes albopictus*^6^. Important reasons for the drastic re-emergence of CHIKV is the expansion of the mosquito vector to urban areas with poor hygiene conditions, progressing climate change as well as the continuous increase in global transportation systems^7^. While other mosquito-borne arboviruses, such as dengue virus (DENV), only cause symptoms in a small fraction of infected individuals, CHIKV infection causes clinical manifestations in approximately 85% of infected individuals^8^. Acute chikungunya fever typically manifests as a rapid-onset illness characterized by fever, (poly)arthralgia, myalgia, maculopapular rashes, headaches and nausea^9^. Notably, 30 to 40% of patients suffer from persistent (poly)arthralgia that develops after clearance of the virus^10^. Appropriately, chikungunya in the African Makonde language translates as “that which bends up”, referring to the debilitating joint pain patients often experience upon CHIKV infection^6^.

To date, the development of an effective treatment for CHIKV infection has not been successful. While various studies reported the development of CHIKV vaccine candidates and antiviral compounds *in vitro* and in animal models, there is no licensed vaccine or therapeutic available to prevent or treat CHIKV infection^6,11–13^. To combat CHIKV, we therefore currently rely on personal protective measures and vector control. The limited resources to control CHIKV infection and the rapid re-emergence emphasize the importance of identifying new compounds that effectively prevent or control CHIKV infection.

Tomatidine is a steroidal alkaloid derived from the stem and leaves of unripe, green tomatoes. It has been described to exhibit a variety of health-beneficial biological activities, including anti-metastatic activity^14^, anti-inflammatory activity^15^, anti-microbial activity^16–18^, and was shown to have a protective effect against age-related muscle atrophy^19^. Tomatidine was also found to exhibit antiviral activity towards the plant viruses Sunnhemp Rosette and Tobacco mosaic virus^20^. We recently identified tomatidine as a novel antiviral compound towards two re-emerging mosquito-borne flaviviruses: dengue virus (DENV) and zika virus (ZIKV)^21^. Potent antiviral activity was seen for all four DENV serotypes and a recent isolate of ZIKV. The most potent effect was seen for DENV serotype 2, with a half maximal effective concentration (EC50) of 0.82 µM. Tomatidine was shown to interfere with various stages of the viral replication cycle of DENV, yet predominantly after virus cell binding and internalization. No antiviral activity was observed for West Nile virus (WNV), a closely related mosquito-borne flavivirus.

Here, we evaluated the antiviral potential of tomatidine towards three different lineages of CHIKV, the East/Central/South African lineage, the original African isolate from 1953 as well as Asian lineage. We observed potent antiviral activity of tomatidine towards the three different CHIKV strains in Huh7 cells, with EC50 and EC90 values between 1.2 µM and 3.8 µM, respectively. Antiviral activity was also observed in Vero-WHO, HFF-1 and U2OS cells. In contrast to DENV, antiviral activity towards CHIKV was specifically seen at post-infection conditions. Tomatidine drastically reduced the number of infected cells and lead to an overall reduction in the number of produced progeny virions. Importantly, its antiviral activity was still observed at 24 hours post-infection, indicating that tomatidine effectively controls at least three rounds of CHIKV replication and highlighting its potential as an antiviral compound to treat CHIKV.

## Results

### Tomatidine inhibits CHIKV infection in various cell lines

**Figure 1:**
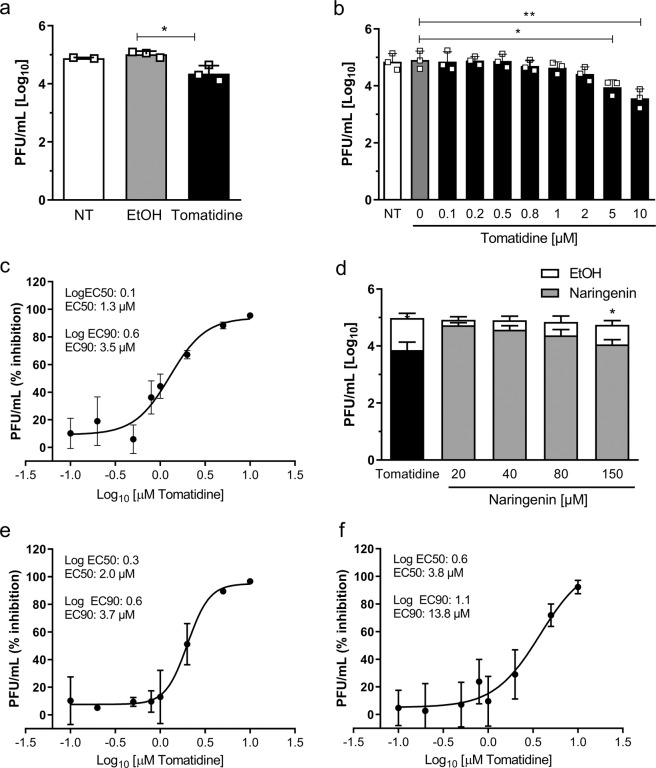
Tomatidine reduces the production of infectious CHIKV particles. (**a**) Antiviral effect of tomatidine on Vero-WHO cells infected with CHIKV-LR at MOI 0.5. Supernatants were collected 9 hpi. (**b–f**) Huh7 cells were infected with distinct CHIKV strains at MOI 1 in presence of increasing concentrations of tomatidine or naringenin or the equivalent volume of EtOH. Supernatants were collected at 9 hpi. (**b,c**) CHIKV-LR OPY1, (**d**) CHIKV-LR OPY1 and naringenin (**e**) CHIKV strain 99659, (**f**) CHIKV strain S27. In all experiments data is represented as mean ± SEM from three independent experiments and differences were assessed with Student’s t-test.

First, we tested the antiviral effect of tomatidine on CHIKV in Vero-WHO cells, as these cells are highly permissive to infection and are often used in related studies^6,22–24^. Prior to the infection experiments, the cytotoxic profile of tomatidine in Vero-WHO cells was determined via an ATPLite assay. As shown in Supplementary Fig. S1a, tomatidine induced a dose-dependent reduction in ATP level with a CC50 value of 149 µM. The CC50 value represents the concentration of tomatidine needed to decrease the ATP level of the cells by 50%. The highest non-toxic tomatidine concentration (defined by survival rates above 75%) was 10 µM (Supplementary Fig. S1a) and was therefore used in subsequent experiments. Vero-WHO cells were incubated with 10 µM tomatidine or the equivalent volume of EtOH and infected with CHIKV-LR at MOI 0.5. Under standard infection conditions, 4.9 ± 0.0 Log infectious virus particles (8.1 × 10^4^ PFU/mL) were produced at 9 hpi (Fig. 1a). A comparable titer was observed for the 0.1% EtOH control (5.0 ± 0.1 Log), indicating that the solvent does not influence infectious virus particle production. In line with previous results on DENV and ZIKV^21^, tomatidine was found to exert significant antiviral activity towards CHIKV. In the presence of 10 µM tomatidine, the infectious virus titer was reduced to 4.4 ± 0.3 Log, which corresponds to a reduction by 76.8% when compared to the EtOH control (Fig. 1a).

In order to validate these findings in a more relevant cell line for human infection, we next investigated the cytotoxic and antiviral potential of tomatidine in Huh7 cells (human hepatic cell line) as hepatocytes are targets during natural CHIKV infection^25^. Furthermore, like with Vero-WHO, these cells are also commonly used in antiviral CHIKV studies^13,26–28^. In our previous study on DENV, the cytotoxicity profile of tomatidine in Huh7 cells has been determined via the MTT assay, measuring the metabolic activity of the cell via mitochondrial activity^21^. Since mitochondrial activity is only one of many factors that determine cell viability, we here performed two additional cytotoxicity assays, the ATPLite assay, which measures the cellular ATP level and a trypan blue staining to detect the number of viable cells after tomatidine treatment. A dose-dependent decrease in ATP levels with increasing tomatidine concentrations was seen. The highest non-toxic tomatidine concentration was 20 µM and the CC50 value was defined as 156 µM (Supplementary Fig. S1b). The highest non-toxic concentration is slightly lower compared to the previously reported results for the MTT assay on Huh7 cells, where the highest non-toxic tomatidine concentration was defined as 30 µM^21^. The trypan blue staining revealed a significant reduction in total cell number at tomatidine concentrations >10 µM (Supplementary Fig. S1c). Based on the above described results, we defined 10 µM as the maximal non-toxic dose of tomatidine, in which metabolic activity, ATP levels and total cell numbers were not significantly changed. To investigate the potential additional cytopathic effect of CHIKV on the viability of tomatidine-treated-Huh7 cells, we next assessed the viability of Huh7 cells in the presence of 10 µM tomatidine under infection conditions via the MTS assay. As displayed in Supplementary Fig. S1d, where the results were normalized to a non-infected, non-treated control, infection with CHIKV at MOI 1 did not have an additional cytopathic effect on the Huh7 cells. Compared to the non-infected, non-treated control, no changes in cell viability were observed upon infection in the presence or absence of 10 µM tomatidine (Supplementary Fig. S1d). Hence, we decided to use 10 µM tomatidine as the highest concentration for all subsequent experiments in Huh7 cells.

To test the antiviral activity of tomatidine in Huh7 cells, cells were incubated with increasing concentrations of tomatidine (0.1 to 10 µM) at the time of infection with CHIKV-LR (MOI 1). On average 4.8 ± 0.2 and 4.9 ± 0.2 Log progeny infectious particles were produced for non-treated and EtOH-treated infection samples, respectively (Fig. 1b). Hence, the presence of EtOH did not influence the production of progeny infectious virus particles. Importantly, a dose-dependent decrease in the production of infectious CHIKV particles was observed in the presence of increasing tomatidine concentrations (Fig. 1b,c). In the presence of 10 µM tomatidine, 3.6 ± 0.2 Log infectious particles were produced, which corresponds to a 95.5% reduction in virus titer when compared to the EtOH control (Fig. 1b,c). Next, the EC50 and EC90 values (i.e. corresponding to 50% and 90% reduction in viral titer, respectively) were calculated by non-linear regression analysis. An EC50 and EC90 value of 1.3 µM and 3.5 µM was found, respectively (Fig. 1c). In order to calculate the SI, the CC50 value determined via ATPLite assay (156 µM) was divided by the EC50 value. Hence, the SI of tomatidine towards CHIKV infection in Huh7 cells is 120.

In order to further validate the antiviral effect of tomatidine towards CHIKV, we next evaluated its antiviral potential in two other human cell lines relevant during natural CHIKV infection, namely, skin fibroblast HFF-1 cells and bone osteosarcoma U2OS cells^28–30^. Similar to above, we again first determined the cytotoxic potential of tomatidine in both cell lines via the ATPLite assay. In both cell lines, a dose-dependent cytotoxic effect was observed, with a CC50 value of 239 µM in HFF-1 cells and 255 µM in U2OS cells (Supplementary Fig. S2a,b). Thereafter, we performed infection experiments at MOI 5 for HFF-1 cells and MOI 1 for U2OS cells in the presence of increasing tomatidine concentrations (0.5 to 10 µM) as described for the Huh7 cells. A higher MOI was used for HFF-1 cells given the relative lower permissiveness of these cells to CHIKV. In HFF-1 cells, a dose-dependent decrease in the number of infectious CHIKV particles was seen with increasing concentrations of tomatidine, with an EC50 value of 2.3 µM and a SI of 104 (Supplementary Fig. S2c). A similar pattern was observed in U2OS cells and led to an EC50 value of 3.9 µM and a SI of 65 (Supplementary Fig. S2d). Altogether, tomatidine exerts a significant antiviral effect towards CHIKV in all cell lines tested, the strongest effect being measured in Huh7 cells.

To compare the antiviral efficacy of tomatidine to another antiviral compound under our experimental settings, we next performed an antiviral study with naringenin, a natural flavonoid that has been reported to have potent antiviral activity towards CHIKV by Ahmadi *et al*. in 2016^24^. To this end, infection experiments were performed in Huh7 cells using four different naringenin concentrations (20–150 µM) to determine the approximate EC50 value. At these concentrations, no cytotoxic effect was measured via the ATPLite assay (Supplementary Fig. S3). At the highest naringenin concentration tested, virus particle production was reduced by 77.6% (4.7 ± 0.3 Log for the EtOH control to 4.1 ± 0.2 Log in treated cells) (Fig. 1d). The approximate EC50 value for naringenin surrounds 40 µM. At this concentration, the CHIKV titer is reduced from 4.9 Log PFU (EtOH control) to 4.6 Log PFU, which corresponds to a reduction by 47%. Hence, in our experimental setup using Huh7 cells, tomatidine is more efficacious at lower concentrations than naringenin.

Next, we tested the antiviral effect of tomatidine towards CHIKV strains from other genotypes, namely the originally isolated African (S27) strain from 1953 and a recently isolated Asian CHIKV genotype (99659) in Huh7 cells^31,32^. For both strains, a strong dose-dependent decrease in virus particle production was observed (Fig. 1e,f). For the Asian strain, the EC50 and EC90 values were 2.0 µM and 3.7 µM, respectively (Fig. 1e). A slightly lower potency was observed towards the African strain, with EC50 and EC90 values of 3.8 µM and 13.8 µM, respectively (Fig. 1f). The respective SI was 78 for the Asian strain and 41.1 for the African strain. Altogether, the above described results demonstrate a potent, dose-dependent antiviral activity of tomatidine towards three distinct CHIKV genotypes in Huh7 cells with comparable EC50 and EC90 values.

### Tomatidine has no direct virucidal effect on the CHIKV particle

**Figure 2:**
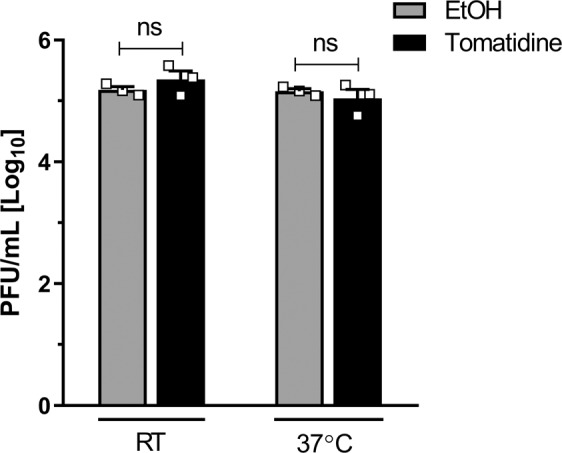
Tomatidine has no direct virucidal effect on CHIKV particles. 2.5 × 10^5^ PFU of CHIKV-LR was incubated with 10 μM tomatidine for 2 h at room temperature (RT) or 37 °C. The infectious titer was determined by plaque assay on Vero-WHO cells. Data is represented as mean ± SEM from three independent experiments and differences were assessed with Student’s t-test.

To test whether the potent antiviral activity of tomatidine towards CHIKV is due to a direct virucidal effect of the compound on the CHIKV particle, 10 µM tomatidine was incubated with 2.5×10^5^ PFU of CHIKV either at room temperature or 37 °C for 2 h. Subsequently, the number of residual infectious CHIKV particles were determined via plaque assay on Vero-WHO cells. The lowest sample dilution used in the plaque assay was 1:10, which corresponds to final tomatidine concentration of 1 µM. At this concentration, we did not detect an antiviral effect of tomatidine in Vero-WHO cells (Supplementary Fig. S4) and therefore we can use the plaque assay on Vero-WHO cells as a readout for virucidal activity. Importantly, incubation of tomatidine with CHIKV alone had no effect on the infectious titer in comparison to the EtOH control (Fig. 2). Thus, the antiviral effect of tomatidine is not owed to a direct virucidal effect on the CHIKV particle.

### Tomatidine reduces CHIKV infection when added up to 6 h post-infection

**Figure 3:**
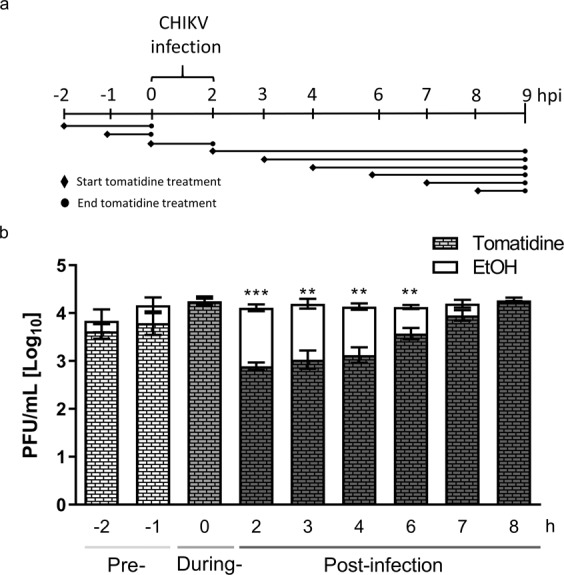
Tomatidine reduces CHIKV infection when added up to 6 hpi. (**a**) Outline of the experimental set-up. (**b**) Huh7 cells were infected with CHIKV-LR at MOI 1 and treated with 10 µM tomatidine or the equivalent volume of EtOH prior to infection (−2 or −1 h), during infection or at different times post-infection (2 to 8 hpi). Supernatants were collected 9 hpi. Data is represented as mean ± SEM from four independent experiments and differences were assessed with Student’s t-test.

To gain a better understanding of the kinetics of the antiviral activity of tomatidine, we next performed a time-of-drug-addition experiment. To this end, Huh7 cells were treated with 10 µM tomatidine pre-, during or post-infection (Fig. 3a). For the pre-treatment conditions, Huh7 cells were incubated with tomatidine for 2 or 1 h before infection. Thereafter, cells were washed to remove tomatidine and infection was initiated. In the during condition, tomatidine was added to the cells at the time of infection. At 2 hpi, inoculum was removed, cells were washed extensively and fresh medium without tomatidine was added. In case of the post-treatment conditions, tomatidine was added to the infected Huh7 cells at 2, 3, 4, 6, 7 or 8 hpi. For all conditions, cells were infected at MOI 1 and supernatants were collected at 9 hpi. Each time-point included a corresponding EtOH control. Whereas for the pre- and during conditions tomatidine did not have any inhibitory effect on progeny infectious virus titers, a significant reduction in infectious particle production was seen when the compound was added at post-infection conditions and up to 6 hpi (Fig. 3b). The strongest effect was observed at the 2 hpi treatment, with a reduction in infectious virus titer by 93.7% when compared to the corresponding EtOH control.

**Figure 4:**
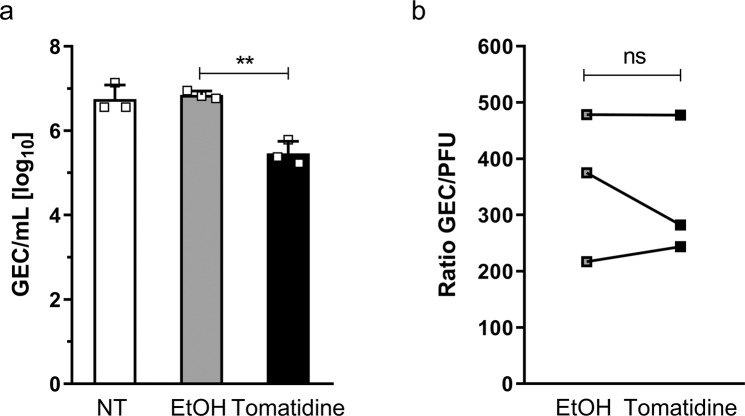
Tomatidine has no effect on the specific infectivity of CHIKV. (**a**) Huh7 cells were infected with CHIKV-LR at MOI 1 and treated with 10 µM tomatidine or the equivalent volume of EtOH at the time of infection. Supernatants were collected at 9 hpi and RT-qPCR was performed to determine GEC/mL. (**b**) Ratio of GEC presented in (**a**) and corresponding PFU determined via plaque assay. Data is represented as mean ± SEM from three independent experiments and differences were assessed with Student’s t-test.

We next investigated whether tomatidine also has an effect on the production of genome-equivalent CHIKV copies (GEC) secreted by infected Huh7 cells. Hereto, Huh7 cells were infected with CHIKV-LR at MOI 1 and treated with 10 µM tomatidine or EtOH at the time of infection. At 9 hpi, the number of GEC was determined. For the non-treated and EtOH control, 6.8 ± 0.2 and 6.9 ± 0.1 Log GEC were detected, respectively (Fig. 4a). In the presence of tomatidine, the number of secreted GEC was reduced to 5.5 ± 0.2 Log GEC (corresponding to a 95.5% reduction) when compared to the EtOH control (Fig. 4a). Subsequent determination of the infectious titer and calculation of the GEC to PFU ratio revealed that there are no significant differences between the ratios in the absence and presence of tomatidine. In presence of EtOH, the ratio was determined as 356 ± 76 and for tomatidine a ratio of 334 ± 72 was calculated (Fig. 4b). Thus, the antiviral effect of tomatidine is related to a reduction in the overall production of GEC rather than specifically interfering with the infectious properties of secreted viruses.

### Tomatidine reduces CHIKV infection in Huh7 cells

**Figure 5:**
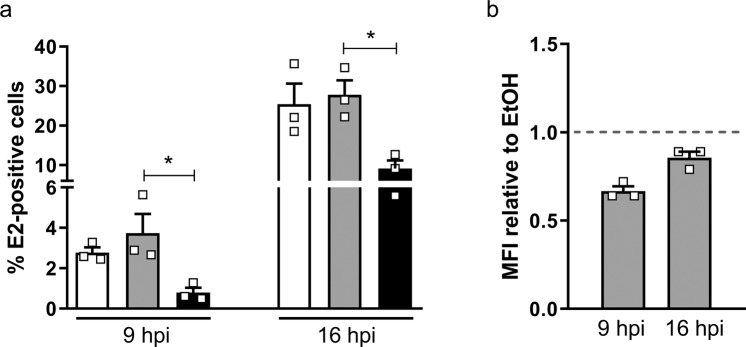
Tomatidine reduces the cell surface expression of the CHIKV E2 protein. Huh7 cells were infected with CHIKV-LR at MOI 1 and treated with 10 µM tomatidine or the equivalent amount of EtOH at the time of infection. (**a**) Cells were collected, fixed and stained for CHIKV E2 protein on the cell surface at 9 and 16 hpi. (**b**) Relative fold changes in MFI in the presence of tomatidine compared to the EtOH control at 9 and 16 hpi. Data is represented as mean ± SEM from three independent experiments and differences were assessed with Student’s t-test.

Based on the findings that tomatidine predominantly acts between 2 and 6 hpi, we next investigated whether tomatidine influences the expression of the CHIKV E2-protein at the cell surface of infected Huh7 cells via flow cytometry. Huh7 cells were infected with CHIKV-LR at MOI 1 and treated with tomatidine or EtOH at the time of infection. In infected cells, the CHIKV E2-protein is translocated to the endoplasmatic reticulum and processed during transit through the secretory pathway prior to expression at the cell surface. Thus, changes within the expression level of E2 at the cell surface in the presence of tomatidine may be indicative for an inhibition of the E2 processing and transport to the cell surface. At 9 hpi, 2.8 ± 0.3% E2-positive cells were observed following infection at MOI 1 (Fig. 5a). At 16 hpi, i.e. after approximately two rounds of CHIKV replication, the number of E2-positive cells increased to 25.4 ± 5.2% (Fig. 5a). Comparable numbers were seen for the corresponding EtOH controls. Importantly, in the presence of tomatidine, the number of E2-positive cells was reduced to 0.8 ± 0.3% and 9.1 ± 2.0% at 9 and 16 hpi, respectively, which corresponds to a reduction by 78.8% (9 hpi) and 67.2% (16 hpi) compared to the EtOH control. Intriguingly, even though a clear reduction in the number of E2-positive cells was seen, a minor effect on the mean fluorescent intensity (MFI) within the E2-positive population was observed (Fig. 5b). At MOI 5, the same pattern was observed (Supplementary Fig. S5). Here, the percentage of infection was reduced by 75.6% (9 hpi) and 53.8% (16 hpi) in the presence of tomatidine, whereas the effect on the MFI was again minor (Supplementary Fig. S5a,b). Comparable results were obtained when cells were permeabilized to allow both intracellular and extracellular staining of E2 (Supplementary Fig. S6a–c). Furthermore, based on the fluorescence intensity measured in both experimental conditions, the vast majority of E2 is expressed at the cell surface at 9 hpi. Taken together, the above results imply that once infection is established, the expression and transport of the E2 protein to the cell surface is mostly unaffected.

### Antiviral activity of tomatidine is sustained for multiple rounds of CHIKV replication

**Figure 6:**
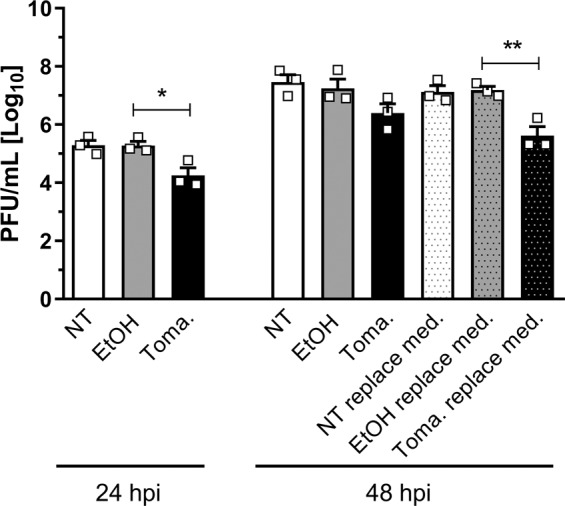
Anti-CHIKV activity of tomatidine is observed for multiple replication cycles. Huh7 cells were infected with CHIKV MOI 0.01 and treated with 10 µM tomatidine at the time of infection. Supernatants were collected 24 and 48 hpi. Alternatively, the medium was replaced by fresh medium containing 10 µM tomatidine or EtOH after 24 h. Data is represented as mean ± SEM from three independent experiments and differences were assessed with Student’s t-test.

During natural infection, the virus replicates continuously within the body. Therefore, we aimed to investigate for how long tomatidine is able to exert its antiviral activity. To this end, we infected cells with CHIKV-LR at MOI 0.01 in the presence of tomatidine and determined progeny virus particle production at 24 and 48 hpi. These time-points correspond to approximately three and six rounds of CHIKV replication, respectively. For samples collected at 48 hpi, the medium remained unchanged or was replaced with fresh medium containing 10 µM tomatidine or EtOH at 24 hpi. Figure 6 reveals that tomatidine reduces CHIKV particle production for at least three rounds of replication. The viral titer of the non-treated samples reached 5.3 ± 0.2 Log progeny infectious particles at 24 hpi (Fig. 6). A similar titer was seen for the EtOH control. Tomatidine treatment lead to a reduction in CHIKV titer by 87.8% to 4.3 ± 0.3 Log compared to the EtOH control (Fig. 6) at 24 hpi. At 48 hpi, no significant difference in infectious virus particle production could be observed between cells treated with EtOH (7.2 ± 0.3 Log) or tomatidine (6.4 ± 0.3 Log) (Fig. 6). Nevertheless, there was still a trend towards a reduction in virus titer in the presence of tomatidine. Importantly, replenishment of tomatidine at 24 hpi did lead to a significant reduction in infectious titer (95.8%) at 48 h (Fig. 6). Similar results were obtained at MOI 0.1, hence at a ten-times higher virus concentration (Supplementary Fig. S7). Here, tomatidine reduced the CHIKV titer by 85.7% at 24 hpi and by 90.6% at 48 hpi with tomatidine replenishment. Altogether, this suggests that tomatidine is able to effectively control CHIKV replication for at least three replication cycles independent of the viral load. To achieve protection at 48 hpi, a replenishment of tomatidine is necessary.

### Tomatidine derivatives solasodine and sarsasapogenin inhibit CHIKV infection

**Figure 7:**
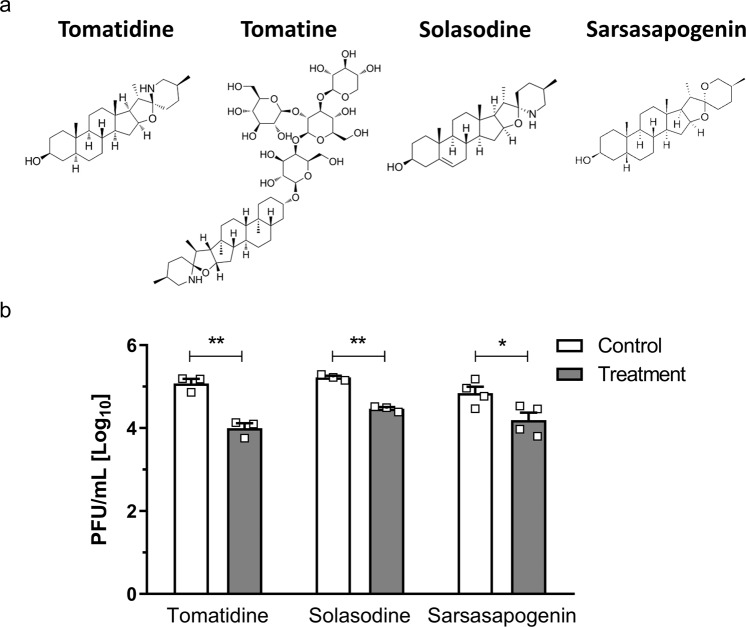
Solasodine and sarsasapogenin show potent antiviral activity towards CHIKV. (**a**) Structures of tomatidine, tomatine, solasodine and sarsasapogenin. (**b**) Antiviral effect of tomatidine and its derivatives on infectious particle production following CHIKV-LR infection at MOI 1. Supernatants were collected 9 hpi. Corresponding treatment concentrations of different compounds: Tomatidine 10 µM, solasodine 5 µM, sarsasapogenin 20 µM. Data is represented as mean ± SEM from three independent experiments except for sarsasapogenin, where four independent experiments were performed, and the mean ± SEM from all four experiments is displayed. Differences were assessed with Student’s t-test.

Testing of structural derivatives of antiviral compounds is a common strategy to enhance their antiviral activity and/or can identify the structural regions of the compound that are relevant for the antiviral activity. We tested three commercially available tomatidine derivatives: tomatine, solasodine and sarsasapogenin for their antiviral effect towards CHIKV-LR in Huh7 cells. The structure of tomatidine and the above derivatives is depicted in Fig. 7a. Based on the cytotoxicity profile (Supplementary Fig. S8a–c), we used a concentration of 5, 5 and 20 µM for tomatine, solasodine and sarsasapogenin in the infectivity assays, respectively. Figure 7b shows that the infectious titer of the non-treated control is 5.02 Log PFU. The EtOH control for each compound showed comparable titers. Unexpectedly however, in presence of CHIKV, tomatine concentrations of 5, 2 and 1 µM lead to a strong cytotoxic effect with extensive cell death through which we were not able to analyze its true antiviral effect. Even at 2 and 1 µM tomatine extensive cell death was observed and therefore we excluded tomatine from further analysis. Solasodine and sarsasapogenin treatment lead to a significant, yet less potent reduction in infectious particle production when compared to the tomatidine control. While for tomatidine-treated cells a reduction to 3.8 Log PFU was observed (92.5% reduction compared to EtOH control), solasodine- and sarsasapogenin-treated cells reduced virus progeny production to 4.5 and 4.2 Log (Fig. 7b). This corresponds to a reduction by 82.5% and 76.4% compared to the EtOH control, respectively. Thus, within the group of tested tomatidine derivatives, tomatidine remains the most potent antiviral compound towards CHIKV.

## Discussion

We have identified the natural steroidal alkaloid tomatidine as a novel antiviral compound towards CHIKV. Tomatidine potently inhibited infection of three different CHIKV genotypes in Huh7 cells. The most potent antiviral effect can be seen towards the CHIKV La Reunion strain, which belongs to the CHIKV East/Central/South African genotype with an EC50 value of 1.3 µM and an SI of 120. For the other tested genotypes, an ancient African genotype and a more recent Asian genotype, EC50 values of 3.8 µM and 2.0 µM were determined, respectively. The corresponding SI are 41.1 (African strain) and 78 (Asian genotype). A direct virucidal effect of tomatidine on the CHIKV particle was excluded. Subsequent time-of-addition experiments demonstrate that the antiviral effect is caused at post-infection conditions and is maintained upon addition of the compound until 6 hpi. Tomatidine did not alter the specific infectivity of CHIKV. Moreover, we showed that tomatidine is able to control CHIKV replication for at least 3 rounds of replication. When testing commercially available structural derivatives of tomatidine, i.e. solasodine and sarsasapogenin, consistent yet slightly less potent antiviral effects towards CHIKV were seen.

The SI is a commonly used parameter in antiviral research to evaluate the specificity of antiviral compounds. The SI index is an adequate general parameter to define the specificity of newly discovered antivirals, however it only gives limited information as it is dependent on the experimental setup, i.e. the MOI and the cell type used. Compared to other reported antiviral compounds towards CHIKV, the SI of tomatidine shows high specificity and potency^11,13,33,34^. Therefore, tomatidine is a promising antiviral compound to treat CHIKV infection.

At the highest non-toxic concentration of tomatidine (10 µM), a 20-fold reduction in viral progeny was observed. Tomatidine was found to act at post-infection conditions as no antiviral effect was observed for the pre- and during infection treatments. Furthermore, a clear (~4-fold) reduction in the number of E2-positive cells in the presence of tomatidine was seen. However, this cannot completely explain the 20-fold reduction observed in the secretion of virus particles, especially since the cells that were infected with CHIKV in presence of tomatidine had substantial expression levels of viral E2. We therefore hypothesize that tomatidine interferes with multiple processes in the replicative cycle of CHIKV. First, infection is aborted after entry and membrane fusion but prior to E2 protein translation and transportation to the cell surface. Second, tomatidine may act on nucleocapsid formation, virion assembly and/or budding of progeny virions. The mode of action of tomatidine might be dependent on the concentration of the compound within the cells. Future studies should reveal the precise mode of action of tomatidine and whether it acts as a direct or host-directed antiviral compound in controlling CHIKV infection.

Recently, we have also demonstrated that tomatidine has a potent antiviral activity towards all four DENV serotypes and ZIKV but not WNV. Intriguingly, all three viruses belong to the flavivirus genus of the family of flaviviridae, and CHIKV, which is a member of the alphavirus genus of the family togaviridae, is much more distantly related to DENV than DENV to WNV. Interestingly, however, by comparing the results for DENV and CHIKV, similarities can be found. First, for both viruses the most potent antiviral effect is seen when tomatidine is added at 2 hpi. This suggests that for both viruses, an early but post-binding and entry step of the virus replication cycle is targeted by tomatidine. For CHIKV, tomatidine only showed effective protection for the post-treatment condition, whereas for DENV the pre and during treatment also showed a clear, albeit less potent, antiviral effect compared to the post-treatment. Therefore, tomatidine may target an additional, early step of the virus replication cycle in DENV infection. Alternatively, the difference between pre- and during treatment condition may also be explained by the differences in the replication time of DENV (24 hours) and CHIKV (8 hours). In this context, tomatidine may be internalized too slowly to exert its antiviral effect towards CHIKV, but not towards DENV. Furthermore, for both viruses the number of cells expressing the viral envelope protein revealed a potent, but much less pronounced antiviral effect compared to the effect seen on the viral particle production again pointing towards a shared mechanism. The question why we do not see an antiviral effect towards WNV, a virus that is much more closely related to DENV and ZIKV, however, remains to be elucidated.

The two out of three commercially available derivatives of tomatidine, solasodine and sarsasapogenin exhibited a constant but less potent antiviral activity compared to tomatidine. These results imply that structural groups altered in the derivatives may be in fact important determinants of tomatidine activity. Solasodine has an additional double bond within the steroidal ring structure, whereas sarsasapogenin is missing the nitrogen of the spiroaminoketal group. Previous studies on the antibacterial properties of tomatidine show that the two extremities of tomatidine, namely the beta-hydroxyl group and the spiroaminoketal group including the basic nitrogen, are responsible for its antibacterial activity^35^. The remaining steroidal rings serve as a structural scaffold. Since sarsasapogenin, which misses the basic nitrogen of tomatidine, shows less potent antiviral activity compared to solasodine and tomatidne, the basic nitrogen in the aminoketal group may be important for the antiviral activity of tomatidine towards CHIKV. Furthermore, and in line with Chagnon *et al*., the double bond within the steroid ring scaffold does not seem to change the antiviral potential of tomatidine. Altogether, these findings suggests that the basic nitrogen may be partly responsible for the antiviral activity of tomatidine. Whether the beta-hydroxyl group also relevant for tomatidine to exert its antiviral effect remains to be evaluated.

In order to further evaluate the potential of tomatidine as an antiviral drug, other important factors including the pharmacokinetic profile, as well as the protein-binding properties of tomatidine have to be taken into account. Unfortunately, to date literature on those aspects is scarce. Tomatidine has been used in several *in vivo* mouse studies and no toxicity was observed up to a concentration of 50 mg/kg^19,36–40^. Only one study measured the steady-state tomatidine plasma levels and revealed a plasma concentration of 287 ng tomatidine per mL after 2 month of oral treatment with 0.05% (w/w) tomatidine added to standard chow^36^. Whereas this study gives some insight into the distribution of tomatidine, further studies are needed to give an in-depth insight into the stability and biodistribution of tomatidine *in vivo*. With regard to protein-binding properties of tomatidine, there is no literature available that directly demonstrates binding of tomatidine to viral or cellular proteins. Nevertheless, numerous papers have demonstrated the ability of tomatidine to modulate different bacterial or host-cell pathways^14,15,40,41^. As an example, a study by Boulet *et al*. in 2017, demonstrated that tomatidine inhibits the *Staphylococcus aureus* ATP Synthase subunit C to exert its antibacterial properties^17^. Moreover, tomatidine has been shown to inhibit cellular ATF4 expression, which leads to a reduction in age-related muscle weakness and atrophy^36^. The ability of tomatidine to control ATF4 expression has also been shown by our recent publication from 2019, though this did not explain the antiviral activity of tomatidine towards DENV^21^. Collectively, despite the numerous functions of tomatidine further studies characterizing the pharmacokinetic profile as well as the protein binding properties of tomatidine are needed to further evaluate tomatidine as a potent antiviral drug.

Our current *in vitro* findings identify tomatidine as a promising antiviral compound to treat CHIKV infection. Toxicity profiles, time-of-addition studies and durability experiments demonstrate a potent and robust antiviral activity. Tomatidine shows a potent antiviral effect when added up to 6 hpi, which is rare among the currently identified potential antiviral compounds towards CHIKV. Nevertheless, further studies regarding the efficacy *in vivo* and the pharmacokinetics of tomatidine are essential to further evaluate its potential as an antiviral compound. Aside from the ability of tomatidine to inhibit CHKV infection, its reported anti-inflammatory activities as well as interferon-stimulating effects may also be of importance as this may alleviate the symptoms associated with CHIKV fever^15,38^.

## Methods

### Cell culture

The human hepatocarcinoma cell line Huh7 (JCRB0403), kindly provided by Tonya Colpitts (University of South Carolina) and the human bone osteosarcoma cell line U2OS (ATCC HTB-96) were maintained in Dulbecco’s minimal essential medium (DMEM) Glutamax (Gibco, The Netherlands) supplemented with 10% FBS (Lonza, Basel, Switzerland), 100 U/mL penicillin and 100 mg/mL streptomycin (PAA Laboratories, Pasching, Austria). The human skin fibroblast cell line HFF-1 (ATCC SCRC-1041) was maintained as described above, with the exception that the medium was supplemented with 15% FBS. Vero-WHO cells, renal cells derived from the African Green monkey, (WHO Reference Cell Bank 10–87, ATCC CCL-81) were maintained in DMEM containing 5% FBS, 100 U/mL penicillin and 100 mg/mL streptomycin. All cell lines were cultured at 37 °C and 5% CO_2_.

### Virus stocks and titration

CHIKV (La Reunion OPY1) was a kind gift from A. Merits (University of Tartu, Estonia). CHIKV strain S27 was kindly provided by S. Guenther, Bernhard-Nocht-Institute for Tropical Medicine and was isolated in 1953 in Tanzania. CHIKV strain 99659, an Asian genotype isolated in 2014 from the British Virgin Islands in the Caribbean was a kind gift from M. Diamond (Washington University, School of Medicine). For virus production, Vero-WHO cells were infected at a multiplicity of infection (MOI) of 0.01 and progeny virions were harvested at 46 h post-infection (hpi). Subsequently, the virus-containing supernatants were centrifuged to clarify from cell debris, aliquoted and stored at −80 °C. Infectious virus titers were determined by plaque assay on Vero-WHO cells and defined as the number of plaque forming units (PFU) per mL. Briefly, Vero-WHO cells were seeded at a density of 13×10^4^ cells/well in a 12-well plate. At 24 h post-seeding, cells were infected with 10-fold serial dilutions of the sample in duplo. At 2 hpi, wells were overlaid with 1% seaplaque agarose (Lonza, Swiss) prepared in 2x MEM. Plaques were counted at 44 hpi. The number of genome-equivalent copies (GEC) per mL was determined via Q-RT-PCR using a StepOne Real-Time PCR instrument (Applied Biosystems, Foster City, CA, USA), as described previously^42^. RNA extraction was performed using a QIAmp viral RNA mini kit (QIAGEN, Venlo, The Netherlands). cDNA synthesis from viral RNA was performed using Omniscript (QIAGEN) and the primers 5′AGCTCCGCGTCCTTTACCA-3′ (forward) and 5′-GCCAAATTGTCCTGGTCTTCCT-3′ (reverse). For qPCR the TaqMan probe 5′-FAMCACTGTAACTGCCTATGCAAACGGCGAC-TAMRA-3′ (Eurogentec, Maastricht, The Netherlands) was used. DNA amplification was performed for 40 cycles of 15 s at 95 °C and 60 s at 60 °C. The number of GEC was determined using a standard curve (correlation coefficient >0.995) of a quantified cDNA plasmid containing the CHIKV E1 sequences (pCHIKV-LS3 1B).

### Chemicals

Tomatidine hydrochloride, solasodine, and sarsasapogenin were purchased from Sigma Aldrich (St. Louis, Missouri, USA) and dissolved in absolute ethanol (EtOH) to a final concentration of 5 mM. Tomatine (Santa Cruz Biotechnology) was prepared in absolute EtOH to a final concentration of 2.5 mM. Naringenin was purchased from Santa Cruz Biotechnology (Dallas, Texas, USA) and dissolved in absolute EtOH to a final concentration of 50 mM. All stock solutions were stored at −20 °C and used for a maximum of 3 months. The final EtOH concentration was below 0.1% in all infection experiments.

### Cytotoxicity assay

The cytotoxicity of tomatine, tomatidine, solasodine and sarsasapogenin was determined *in vitro* via an ATPLite Luminescence Detection assay system. Huh7, U2OS, HFF-1 or Vero-WHO cells were seeded in a white polystyrene 96-well plate at a density of 8.0×10^3^. At 24 h post-seeding, cells were treated with increasing concentrations of the different compounds ranging from 1 to 500 µM. At 24 h post-treatment, 50 µL mammalian cell lysis solution was added per well and the plate was incubated for 5 min at room temperature on an orbital shaker. Subsequently, 50 µL substrate solution was added to wells and the plate was incubated for 5 min, as before. Then, the plate was incubated for 10 min in the dark. Luminescence was measured with a microplate reader (Biotek, Sinergy, HT, Vermont, USA). Cytotoxicity was expressed as follows:

$$ \% \,{Cytotoxicity}=\frac{({\rm{Abs}}\,{\rm{sample}})-({\rm{Abs}}\,{\rm{blank}})}{({\rm{Abs}}\,{\rm{negative}}\,{\rm{control}})-({\rm{Abs}}\,{\rm{blank}})}\ast 100$$

For tomatidine, cytotoxicity in Huh7 cells was also measured via determining the total cell number. To this end, Huh7 cells were seeded in a 12-well plate at a density of 1.2 × 10^5^ cells/well. At 24 h post-treatment, cells were trypsinized with 1x Trypsin/EDTA (Gibco), stained with trypan blue (Sigma) and counted manually using a Buerker counting chamber depth 0.100 mm (Marienfeld, Lauda-Koenigshofen, Germany).

Additionally, the cytotoxic effect at the highest non-toxic concentration of tomatidine (10 µM) was evaluated in Huh7 cells in the presence of CHIKV via the CellTiter 96 Aqueous Non-Radioactive Cell Proliferation Assay (MTS assay). To this end, Huh7 cells were seeded in a 96-well plate as described above and infected with CHIKV-LR at MOI 1. At the time of infection, cells were treated with 10 µM tomatidine or the equivalent volume of EtOH. At 24 h post infection, 20 µL MTS/PMS solution was added per well and the plate was further incubated for 2 h at 37 °C. Subsequently, 10% formaldehyde was added to inactivate the virus and the absorbance was measured at 490 nm with a microplate reader. Cytotoxicity was expressed as described for the ATPLite assay. Values were then displayed as percentage compared to a non-infected, non-treated control.

### Antiviral assays

Vero-WHO, Huh7, U2OS and HFF-1 cells were infected with CHIKV at MOI 0.5, 1 or 5. Unless indicated otherwise, tomatidine, solasodine, sarsasapogenin or naringenin (or the equivalent volume of ethanol) were added at the time of infection. The virus inoculum was removed at 2 hpi, after which the cells were washed three times, and compound-containing medium was added until sample collection. For the time-of-addition experiments, Huh7 cells were treated with tomatidine either pre-, during or post-infection. For the pre-treatment, 10 µM tomatidine was added 1 or 2 h before infection, as indicated. At the time of infection, cells were washed three times before the addition of the virus inoculum. For the during condition, tomatidine was added together with the virus inoculum and was present for only for 2 h. For the post-treatment condition, tomatidine was added at 1, 2, 4, 6, 7 and 8 hpi. In all experiments, the virus inoculum was removed after two hours and the supernatant was collected at 9 or 16 hpi.

### Virucidal effect

CHIKV-LR (2.5×10^5^ PFU) was incubated in the presence or absence of 10 µM tomatidine or the equivalent volume of ethanol at room temperature or 37 °C for 2 h in a total volume of 250 µL. Subsequently, the infectious virus titer was determined via plaque assay.

### Flow cytometry

Huh7 cells were trypsinized with 1x Trypsin/EDTA (Gibco) and fixed with 2% paraformaldehyde. After fixation, the cells were stained with a rabbit anti-E2-stem antibody (obtained from G. Pijlman, Wageningen University, Wageningen, The Netherlands) diluted 1:1000 and a AF647 conjugated chicken anti-rabbit antibody diluted 1:300 (Life Technologies, Carlsbad, California, USA). Flow cytometry analysis was performed with a FACSCalibur cytometer (BD Biosciences) and analysis was performed via Kaluza 1.1.

### Durability study

Huh7 cells were infected with CHIKV-LR OPY1 at MOI 0.01 or MOI 0.1 and treated with 10 µM tomatidine or EtOH, as described in the antiviral assay section. Supernatants were collected at 24 or 48 hpi. For the samples collected at 48 hpi, the medium remained unchanged after infection or was replaced by fresh complete medium containing 10 µM tomatidine or EtOH at 24 hpi. Virus titer was determined via plaque assay.

### Statistical analysis

The EC50 and EC90 values represent the tomatidine concentration that reduces virus particle production by 50 or 90%, respectively. The CC50 and CC90 values, represent the tomatidine concentration that causes 50 and 90% cytotoxicity, respectively. The corresponding dose-response curves were fitted by non-linear regression analysis using a sigmoidal model. The calculated selectivity index (SI) represents the ratio of CC50 to EC50. All data was analyzed with GraphPad Prism (La Jolla, CA, USA) and data are presented as mean ± SEM. Statistical differences were evaluated via Student’s t-test, a value of p ≤ 0.05 was considered significant, with *p ≤ 0.05, ** p ≤ 0.01 and *** p ≤ 0.001.

## Supplementary information

Supplementary Dataset 1.

## Data Availability

The data described in this study are available from the corresponding author upon request.
