# Supplementary material for: Tomatidine, a natural steroidal alkaloid shows antiviral activity towards chikungunya virus in vitro
Source: Sci Rep. 2020 Apr 14;10:6364. doi: 10.1038/s41598-020-63397-7 (PMC7156627; doi:10.1038/s41598-020-63397-7)
Supplement: Supplementary file 1 — Supplementary Dataset 1. [file 41598_2020_63397_MOESM1_ESM.pdf]

Tomatidine, a natural steroidal alkaloid shows antiviral activity towards chikungunya virus in vitro

Berit Troost[a], Lianne M. Mulder[a], Mayra Diosa-Toro[a,b], Denise van de Pol[a], Izabela Rodenhuis-Zybert[a], Jolanda M. Smit[a*]

a. Department of Medical Microbiology and Infection Prevention, University of Groningen; University Medical Center Groningen; Groningen, the Netherlands.
b. Present address: Programme in Emerging Infectious Diseases, Duke-NUS Medical School, Singapore 169857

* Corresponding author: Jolanda M. Smit
PO Box 30.001, EB88
9700 RB Groningen, The Netherlands.
E-mail: jolanda.smit@umcg.nl
Phone number: 310503616562

Supplementary Information

Supplementary Fig. S1. Tomatidine does not show cytotoxicity up to a concentration of 10 μM in Huh7 and Vero-WHO cells.

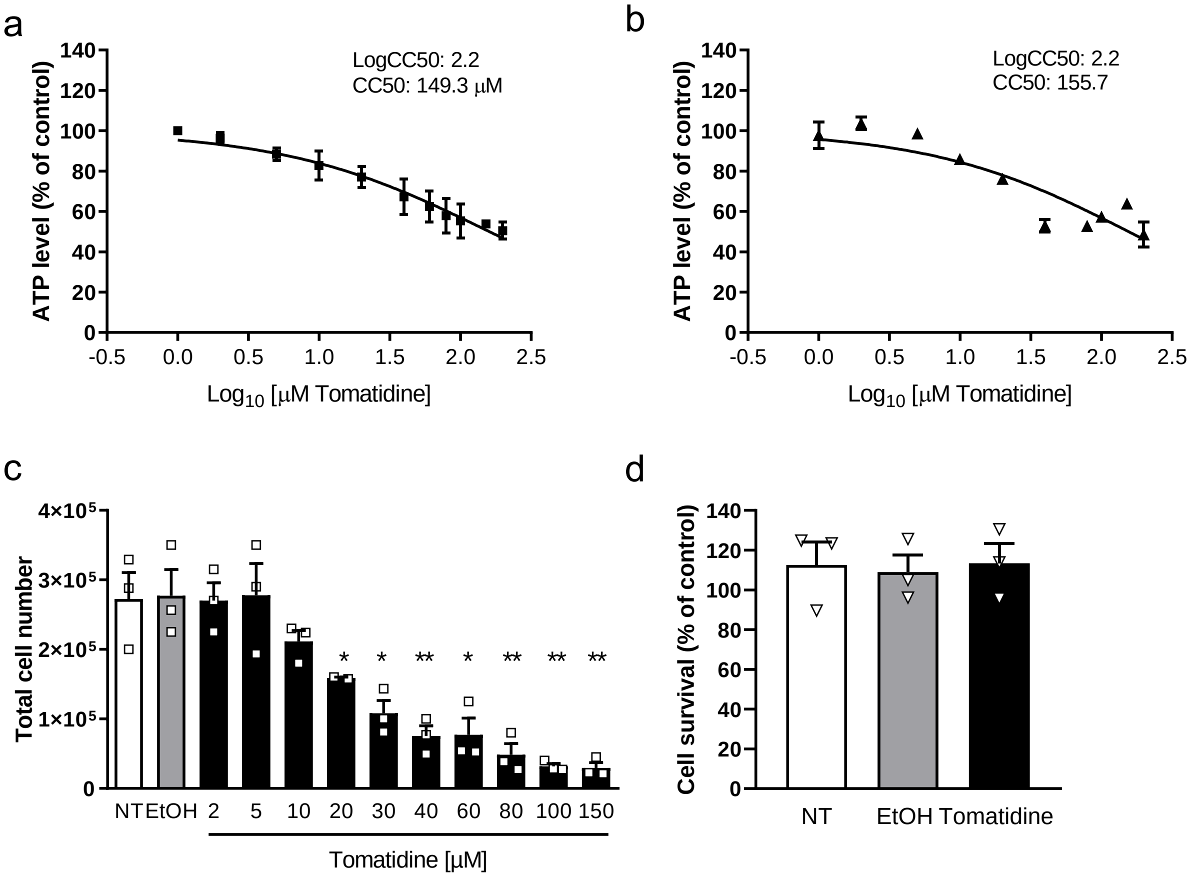

Supplementary Fig. S2. Cytotoxic and antiviral effect of tomatidine towards CHIKV in HFF-1 and U2OS cells.

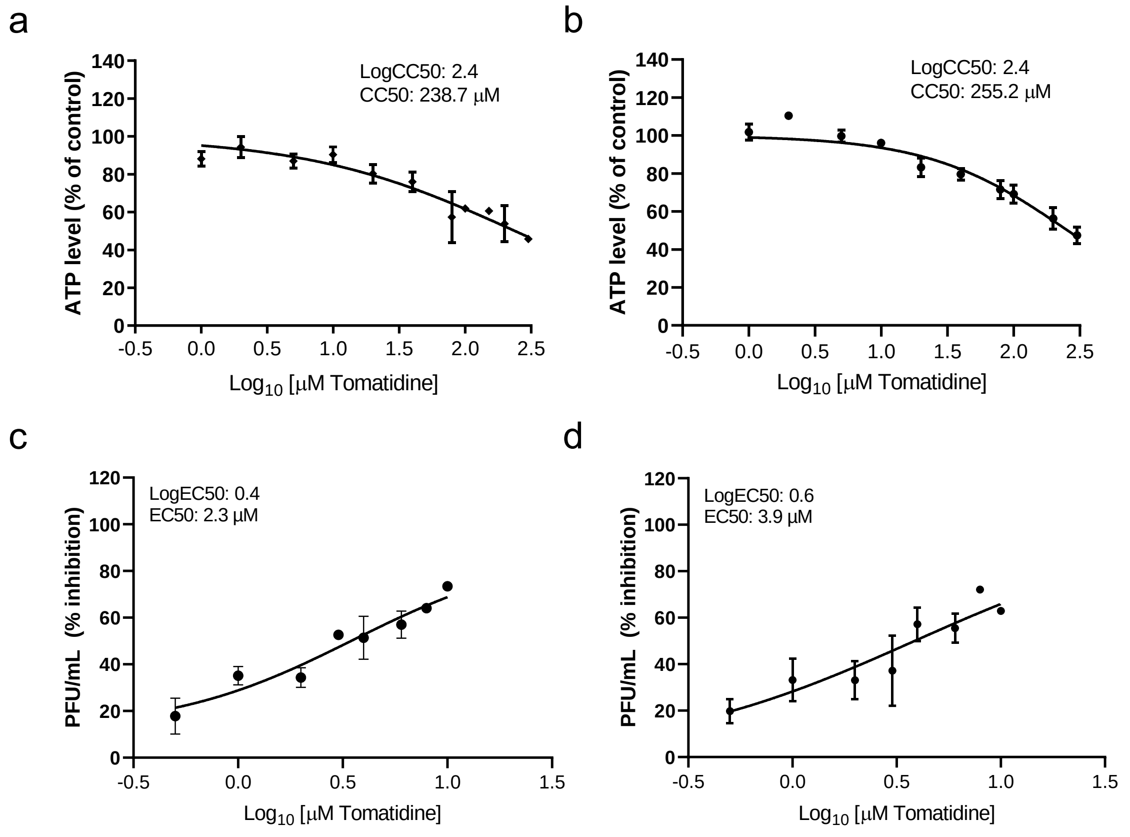

Supplementary Fig. S3. Cytotoxic effect of naringenin on Huh7 cells.

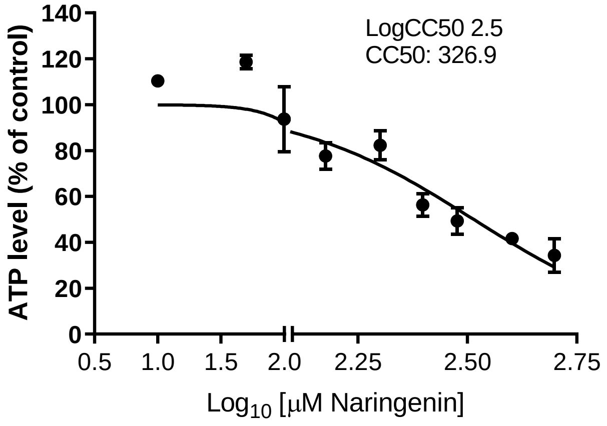

Supplementary Fig. S4. Effect of tomatidine on CHIKV infectious particle production in Vero-WHO cells.

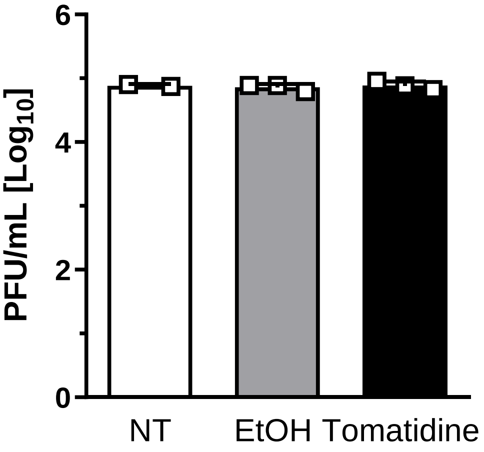

Supplementary Fig. S5. Tomatidine reduces the cell surface expression of the CHIKV E2 protein.

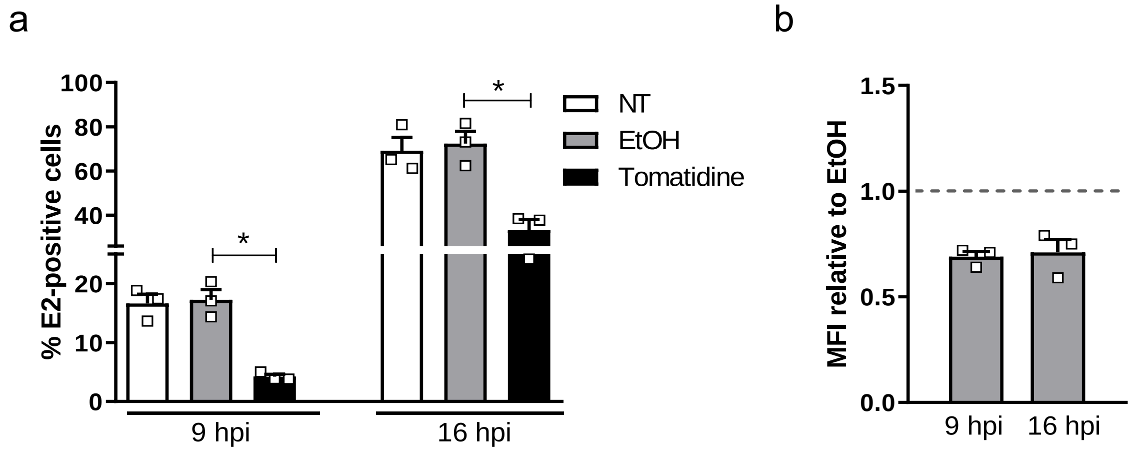

Supplementary Fig. S6. Tomatidine reduces the cellular expression of the CHIKV E2 protein.

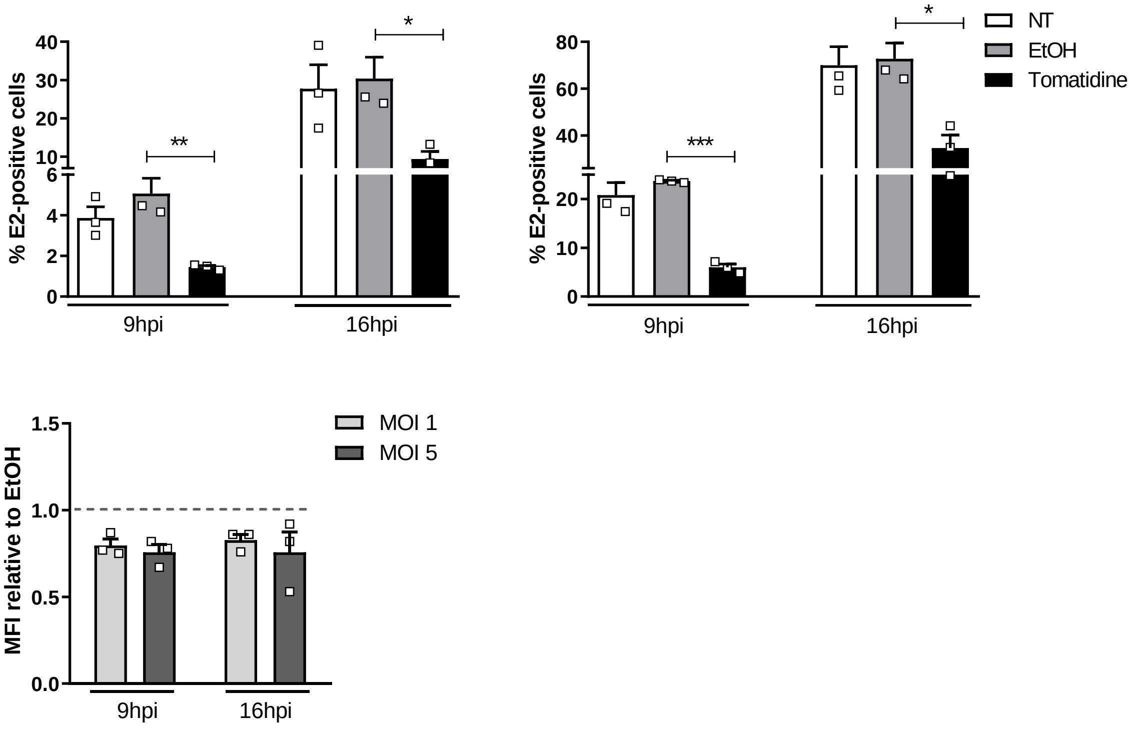

Supplementary Fig. S7. Anti-CHIKV activity of tomatidine is observed for multiple replication cycles.

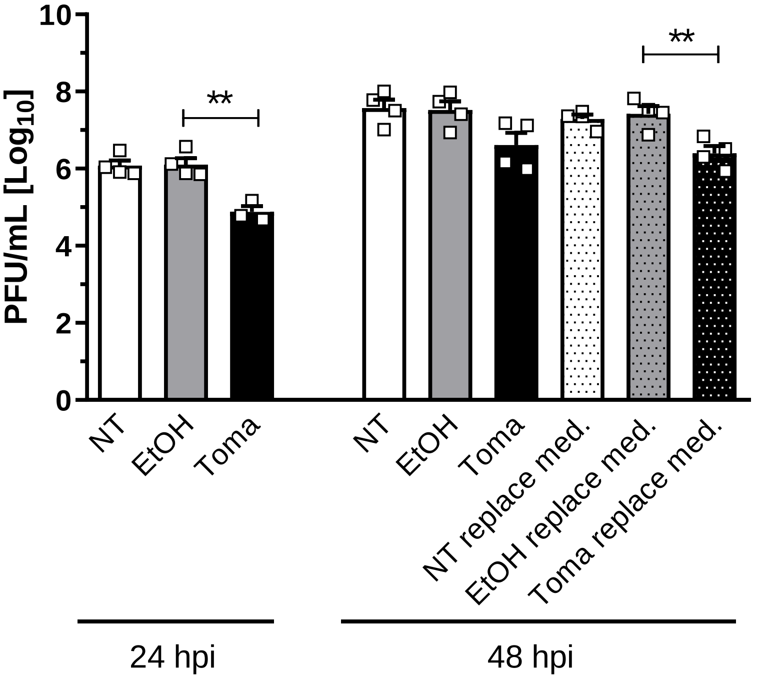

Supplementary Fig. S8. Cytotoxic effect of tomatidine derivatives on Huh7 cells.

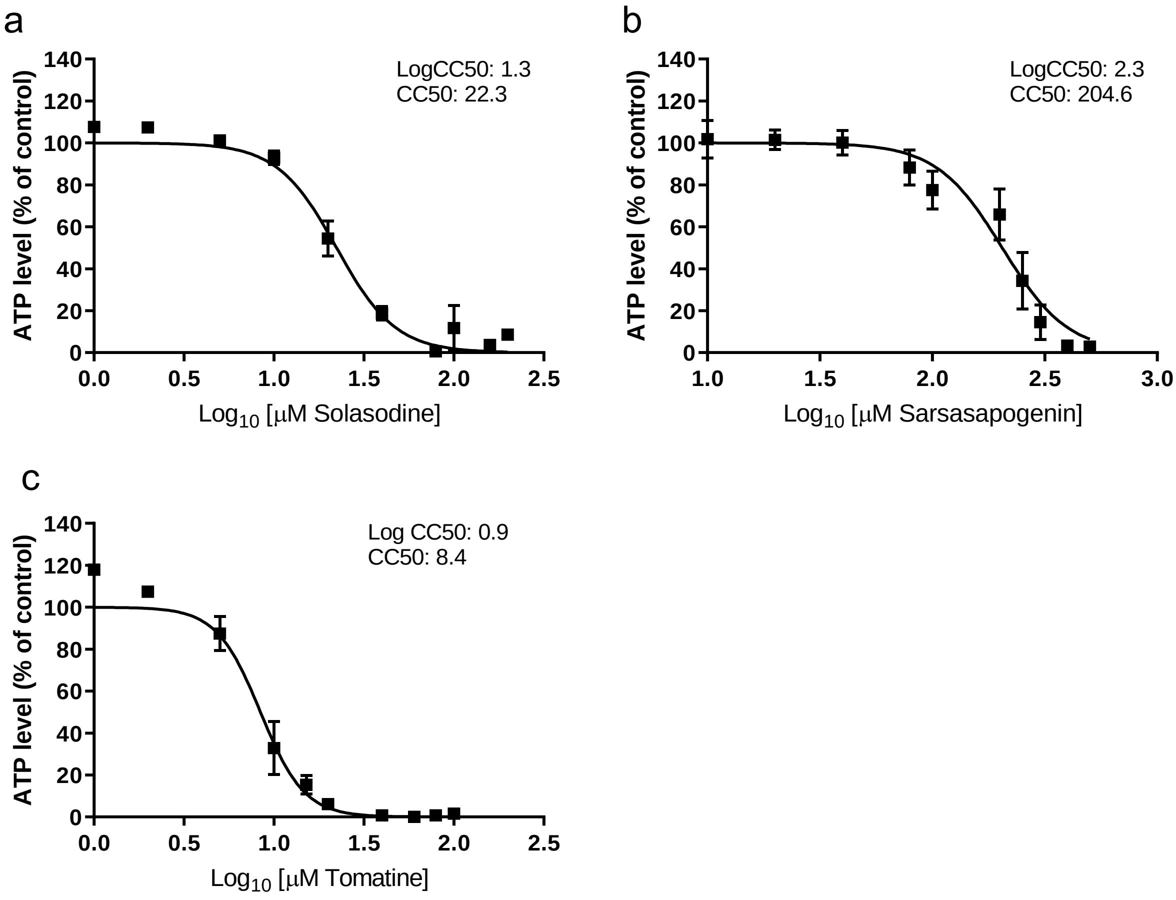

Caption for Figures:

Supplementary Figure S1. Tomatidine does not show cytotoxicity up to a concentration of 10 μM in Huh7 and Vero-WHO cells. (a) Dose-response curve of the ATP level in Vero-WHO cells assessed by ATPLite assay at 24 h in the presence of increasing tomatidine concentrations. (b-c) Huh7 cells were treated with increasing concentrations of tomatidine for 24 h. (b) ATPLite assay was performed to determine the ATP level relative to the EtOH control. (c) Trypan blue staining determine the total cell number after 24 h. (d) Huh7 cells were infected with CHIK-LR at MOI 1 and treated with 10 μM tomatidine or the equivalent volume of EtOH at the time of infection. Cell viability was assessed via the MTS assay 24 hpi. Viability is expressed as percentage compared to a non-infected, non-treated control. Data is represented as mean ± SEM from three independent experiments and differences were assessed with Student's t-test.

Supplementary Figure S2. Cytotoxic and antiviral effect of tomatidine towards CHIKV in HFF-1 and U2OS cells. (a-b) Dose-response curve of the ATP level in (a) HFF-1 and (b) U2OS cells assessed by ATPLite assay at 24 h in the presence of increasing tomatidine concentrations. (c) HFF-1 cells were infected with CHIK-LR at MOI 5 and treated with increasing tomatidine concentrations at the time of infection. Supernatants were collected 16 hpi. (d) U2OS cells were infected with CHIK-LR at MOI 1 and treated with increasing tomatidine concentrations at the time of infection. Supernatants were

collected 9 hpi. Data is represented as mean ± SEM from three independent experiments and differences were assessed with Student's t-test.

Supplementary Figure S3. Cytotoxic effect of naringenin on Huh7 cells. Dose-response curve of the ATP level in Huh7 cells assessed by ATPLite assay at 24 h in the presence of increasing naringenin concentrations. Data is represented as mean ± SEM from three independent experiments and differences were assessed with Student's t-test.

Supplementary Figure S4. Effect of tomatidine on CHIKV infectious particle production in Vero-WHO cells. Vero-WHO cells were infected with CHIKV-LR at MOI 0.5 in the presence of 1 µM tomatidine or the equivalent volume of EtOH. Virus titer was determined via plaque assay. Data is represented as mean ± SEM from three independent experiments and differences were assessed with Student's t-test.

Supplementary Figure S5. Tomatidine reduces the cell surface expression of the CHIKV E2 protein. Huh7 cells were infected with CHIKV-LR at MOI 5 and treated with 10 µM tomatidine or the equivalent amount of EtOH at the time of infection. (a) Cells were collected, fixed and stained for CHIKV E2 protein on the cell surface at 9 and 16 hpi. (b) Relative fold changes in MFI in the presence of tomatidine compared to the EtOH control at 9 and 16 hpi. Data is represented as mean ± SEM from three independent experiments and differences were assessed with Student's t-test.

Supplementary Figure S6. Tomatidine reduces the cellular expression of the CHIKV E2 protein. Huh7 cells were infected with CHIKV-LR at MOI 1 (a) and MOI 5 (b) and treated with 10 µM tomatidine or the equivalent amount of EtOH at the time of infection. Cells were collected, fixed, permeabilized and stained for cellular CHIKV E2 protein at 9 and 16 hpi. (c) Relative fold changes in MFI in the presence of tomatidine compared to the EtOH control at 9 and 16 hpi. Data is represented as mean ± SEM from three independent experiments and differences were assessed with Student's t-test.

Supplementary Figure S7. Anti-CHIKV activity of tomatidine is observed for multiple replication cycles. Huh7 cells were infected with CHIKV at MOI 0.1 and treated with 10 µM tomatidine at the time of infection. Supernatants were collected 24 and 48 hpi. Alternatively, the medium was replaced by fresh medium containing 10 µM tomatidine or EtOH after 24 h. Data is represented as mean ± SEM from three independent experiments and differences were assessed with Student's t-test.

Supplementary Figure S8. Cytotoxic effect of tomatidine derivatives on Huh7 cells. Dose-response curve of the ATP level in Huh7 cells treated with increasing concentrations of (a) solasodine, (b) sarsasapogenin and (c) tomatine assessed by ATPLite assay at 24 h. Data is represented as mean ± SEM from three independent experiments.
